# Optimization of an *in vitro* drug screening assay for anti-*Trypanosoma cruzi* activity using epimastigotes

**DOI:** 10.1093/biomethods/bpag051

**Published:** 2026-09-08

**Authors:** Jhindy-Tatiana Perez, María-Helena Arias, Giovanny Garavito

**Affiliations:** Universidad Nacional de Colombia, Sede Bogotá, Facultad de Ciencias, Departamento de Farmacia, Grupo de Investigación FaMeTra (Farmacología de la Medicina Tradicional y Popular), Carrera 30 45-03, Bogotá D.C. 111311, Colombia; Universidad Nacional de Colombia, Sede Bogotá, Facultad de Ciencias, Departamento de Farmacia, Grupo de Investigación FaMeTra (Farmacología de la Medicina Tradicional y Popular), Carrera 30 45-03, Bogotá D.C. 111311, Colombia; Universidad Nacional de Colombia, Sede Bogotá, Facultad de Ciencias, Departamento de Farmacia, Grupo de Investigación FaMeTra (Farmacología de la Medicina Tradicional y Popular), Carrera 30 45-03, Bogotá D.C. 111311, Colombia

**Keywords:** *Trypanosoma cruzi*, epimastigotes, parasitic sensitivity tests, Chagas disease, neglected tropical diseases, drug discovery

## Abstract

Chagas disease, caused by *Trypanosoma cruzi*, poses a significant global health burden. Current treatment relies solely on benznidazole and nifurtimox, both with limited efficacy and significant adverse effects. *In vitro* phenotype-based screening enables identification of alternative treatments. While epimastigote-based screening is not suitable for primary drug discovery, it provides valuable complementary data for compound evaluation. However, the lack of standardized experimental conditions across studies compromises reproducibility and inter-laboratory comparability. Here, we optimized experimental conditions for *T. cruzi* epimastigote drug susceptibility assays. *Trypanosoma cruzi* epimastigotes (strain Y) were cultured under systematically varied conditions to optimize medium composition, serum supplementation, medium replacement frequency, inoculum density, and treatment exposure time. Epimastigote growth peaked on day 8, declining thereafter. Low initial parasite inoculum showed reduced growth and high variability, while high parasite densities formed aggregates. Growth rates were similar across media, but Roswell Park Memorial Institute medium could promote trypomastigote-like forms. Fetal bovine serum (FBS) enhanced growth rates, with a positive correlation between serum concentration and proliferation. Three-day medium replacement maintained >98% epimastigotes. Benznidazole exhibited an IC_50_ of 16.61 ± 0.38 µM under optimized conditions. Optimal conditions include: Liver Infusion Tryptose or Brain Heart Infusion medium with 10% FBS, medium replacement every 3 days, 1 × 10^6^ parasites/ml inoculum, and 72-h treatment at 28°C. The assay demonstrated robustness across microplate designs, producing consistent results with both U-bottom and flat-bottom 96-well plates. Under these optimized conditions, benznidazole serves as an effective positive control.

## Introduction

Chagas disease (CD), also known as American trypanosomiasis, is caused by the parasite *Trypanosoma cruzi* and is classified by the World Health Organization (WHO) as a neglected tropical disease. The disease is primarily transmitted through the feces of infected triatomine insects [1]. Nearly 8 million people are currently infected, with 65–100 million at risk worldwide. CD poses several critical challenges: it has emerged as an infectious disease threat in previously non-endemic areas due to human migration and climate-change-driven vector displacement, leading to new outbreaks and alternative transmission routes [2, 3]; the acute phase is often asymptomatic, resulting in widespread underdiagnosis; and the most serious cardiac complications typically develop years to decades after the initial infection [2].

Treatment options for CD remain severely limited to two drugs: benznidazole and nifurtimox. Both carry significant limitations, including severe adverse effects and limited efficacy in the chronic phase, with cure rates of only 20%–60%. Because CD primarily affects impoverished and marginalized communities, pharmaceutical companies lack sufficient financial incentive to invest in new therapeutic options [4].

*In vitro* investigations have recently demonstrated promising trypanocidal activity for novel compounds. However, translation of these findings into clinical applications has been largely unsuccessful, with only six substances—allopurinol, itraconazole, posaconazole, fluconazole, fosravuconazole, and fexinidazole—advancing to clinical studies. This limited progress stems primarily from the absence of standardized protocols and consensus regarding experimental conditions in *in vitro* pharmacological models. Protocols vary markedly across studies, and specific experimental conditions are frequently unreported. This methodological inconsistency is particularly problematic because these *in vitro* studies serve as the primary screening mechanism for identifying candidate active pharmaceutical ingredients (APIs) [5].

The lack of harmonized *in vitro* pharmacological models poses two major challenges: first, it prevents meaningful comparison across studies; and second, it complicates decisions regarding which compounds merit advancement to *in vivo* testing and clinical trials [5–9]. This critical gap ultimately contributes to the low success rate of compounds in clinical trials. Therefore, establishing standardized bioassay practices and well-defined experimental conditions is essential to generate rigorous proof of concept in the drug discovery process for CD [7].

Although epimastigotes represent the replicative stage found in the insect vector and differ markedly from intracellular amastigotes in morphology, host environment, and metabolic flux [10, 11], they retain core biochemical systems relevant to parasite survival, including glycosomal metabolic pathways, the trypanothione-dependent redox network, and enzymatic systems involved in the activation of nitroheterocyclic drugs. Glycosomes contain enzymes involved in glycolysis and other essential metabolic pathways, and several glycosomal enzymes have been validated as potential drug targets in trypanosomatids [12]. Similarly, trypanothione-based antioxidant metabolism is a central component of *T. cruzi* redox homeostasis, contributing to peroxide detoxification and parasite survival [13]. The pharmacological relevance of these systems is further illustrated by benznidazole, whose trypanocidal activity depends on parasite-encoded type I nitroreductase-mediated activation [14].

Modern *T. cruzi* drug discovery relies on tiered screening cascades that combine assays with different levels of biological complexity to balance throughput, parasite-stage relevance, selectivity, and mechanistic resolution [15]. In this context, a standardized epimastigote assay can provide a streamlined, scalable, and reproducible approach for evaluating compound collections under defined experimental conditions, thereby complementing more complex intracellular models. Its value lies in facilitating the identification and prioritization of compounds with antiparasitic activity while reducing methodological variability at an early stage of the drug discovery process. This is particularly relevant when compound libraries have undergone prior physicochemical or computational filtering for drug-likeness, although such preselection does not establish intracellular exposure, host-cell selectivity, or activity against mammalian-stage parasites. Compounds showing activity in epimastigote assays can therefore be integrated into subsequent evaluation using intracellular amastigote models and, where warranted, *in vivo* studies, allowing activity, selectivity, and stage-specific responses to be progressively characterized within a complementary, multistage drug discovery framework [1, 16].

An optimized phenotype-based *in vitro* assay for screening anti-*T. cruzi* compounds is critically needed. Such an assay must be reliable, straightforward to implement, and accessible using standard laboratory equipment. This study systematically optimized key conditions for an *in vitro* pharmacological assay designed to evaluate compounds with potential activity against *T. cruzi* in epimastigotes. This work addresses the methodological inconsistencies that currently limit drug discovery efforts and provides a foundation for standardized screening practices in the field.

## Materials and methods

### Pharmacological model of *in vitro* culture of *T. cruzi* epimastigotes

*Trypanosoma cruzi* Y strain was obtained from the *Laboratorio de Investigaciones en Parasitología Tropical de la Universidad del Tolima*. Human serum (HS) was provided by the *Instituto Distrital de Ciencia, Biotecnología e Innovación en Salud* (IDCBIS), while fetal bovine serum (FBS) was purchased from Gibco (Gibco® Thermo Fisher Scientific, Waltham, USA). Three culture media were used: Liver Infusion Tryptose (LIT), Roswell Park Memorial Institute (RPMI)—1640, and Brain Heart Infusion (BHI) (Gibco® Thermo Fisher Scientific, Waltham, USA). All media were prepared, adjusted to pH 7.5, and stored at 4°C. The reference drug, Benznidazole, was purchased from Sigma-Aldrich® (Merck KGaA, Darmstadt, Germany).

Trypomastigotes were obtained from blood samples of infected mice following Lourenço *et al*. [17] with modifications. Swiss ICR mice (23 ± 1 g) were infected intraperitoneally with 1 × 10^5^ parasites/ml. At peak parasitemia, mice were anesthetized, and 1 ml of blood was collected via cardiac puncture. The blood was centrifuged at 500 × g for 30 min. Plasma was replaced with 5 ml of LIT medium supplemented with 10% fetal bovine serum (FBS) and 1% hemin. After a second centrifugation, the medium was discarded, and the infected red blood cells were resuspended in 5 ml of LIT medium supplemented with 10% FBS in a culture tube. The tube was incubated at 28°C with agitation every 48 h. On day 10, transformation from trypomastigotes to epimastigotes was confirmed by microscopy. The culture medium was replaced, and incubation continued until day 15 to complete the transformation.

Baseline conditions for epimastigote culture were established according to a scoping review [18], comprising incubation at 28°C, LIT medium supplemented with 10% FBS, and antibiotic-free conditions. Parasite density was monitored by Neubauer chamber counts. Statistical data analysis was performed using RStudio Desktop version 1.4.1717. Ethical approval: Faculty of Sciences, UNAL (Acta 14-2017).

### Conditions to optimize

Optimization parameters were selected based on their critical influence on reproducibility, sensitivity, and practical applicability to both biological models and pharmacological assays. The following variables were systematically evaluated: culture medium type and serum supplementation, medium replacement frequency, initial parasite density, treatment exposure time, and microplate format (U-bottom versus flat-bottom 96-well plates).

### Parasite growth kinetics

Two independent experiments were conducted under the baseline conditions described above. Cultures were initiated at 1 × 10^6^ parasites/ml, a concentration widely used in the literature [18]. Parasite density was assessed daily for 11 days using a Neubauer hemocytometer. All experiments included three biological replicates, and results are expressed as mean ± standard deviation.

### Optimization of culture medium, serum supplementation, and initial infectious inoculum

A multifactorial design was used to optimize key culture conditions for *T. cruzi* epimastigotes. Four factors were evaluated: culture medium type, serum type, serum percentage, and initial parasite inoculum (parasites/ml). Their levels were systematically combined to assess effects on parasite proliferation (Table 1), yielding 54 distinct factor combinations in total.

**Table 1:** Variables and their levels used in the factorial design.

Variable	Media	Supplementation	Serum supplementation (%)	Density (parasites/ml)
Levels	LIT	FBS	10	1 × 10^5^
	BHI	HS	20	1 × 10^6^
	RPMI		30	5 × 10^6^

### Culture media replacement frequency

The effect of media replacement frequency on *T. cruzi* epimastigote growth and differentiation was assessed using glass culture tubes at an initial concentration of 1 × 10⁶ parasites/ml. Replacement was performed by adding 5 ml of fresh medium to 3 ml of existing culture. Four frequencies were tested: every 3, 7, or 10 days, and no replacement (control). At each designated time point, parasite density and morphological differentiation were evaluated, with differentiation measured as the epimastigote-to-trypomastigote ratio.

### Pharmacological test for antiparasitic activity against *T. cruzi* epimastigotes

#### Treatment exposure time

The optimal treatment exposure time for antiparasitic compound evaluation was determined from *T. cruzi* growth kinetics under the optimized culture conditions. Parasite density was assessed at 24-h intervals over 11 days to identify the time point that balanced sufficient parasite multiplication with minimal spontaneous differentiation, ensuring assay reliability.

#### Culture well plates

The influence of microplate format on *T. cruzi* epimastigote multiplication was assessed using an 11-day growth kinetics assay comparing U-bottom and flat-bottom 96-well plates under the optimized experimental conditions established in this study. For each day evaluated, triplicate wells were allocated and sacrificed for parasite enumeration. No medium replacement was performed during this period, thereby preventing mechanical disruption of the parasite suspension during sampling.

#### Reference substances and controls

A concentration-response assay was conducted under optimized conditions. Benznidazole served as the reference drug at concentrations ranging from 0.4 to 30.7 µM. Two controls were included: a vehicle control with 0.04% DMSO (showing no antiparasitic activity) and a growth control with LIT medium supplemented with 10% FBS. Each treatment group was tested in five experimental units across two independent replicates. Parasite density was assessed using a Neubauer hemocytometer, and IC_50_ values were calculated by regression analysis.

## Results

### Growth kinetics of *T. cruzi* epimastigotes

Growth kinetics of *T. cruzi* epimastigotes over 11 days was best described by a second-order polynomial model, reflecting a growth-and-decline pattern (*R*² = 0.9847), which showed a slightly higher goodness-of-fit than the linear model (*R*² = 0.9792) and other models evaluated (Fig. 1A). Kinetic data showed proliferative (logarithmic) growth phase between days 1 and 8, followed by the onset of the stationary phase from day 9 onward.

**Figure 1:**
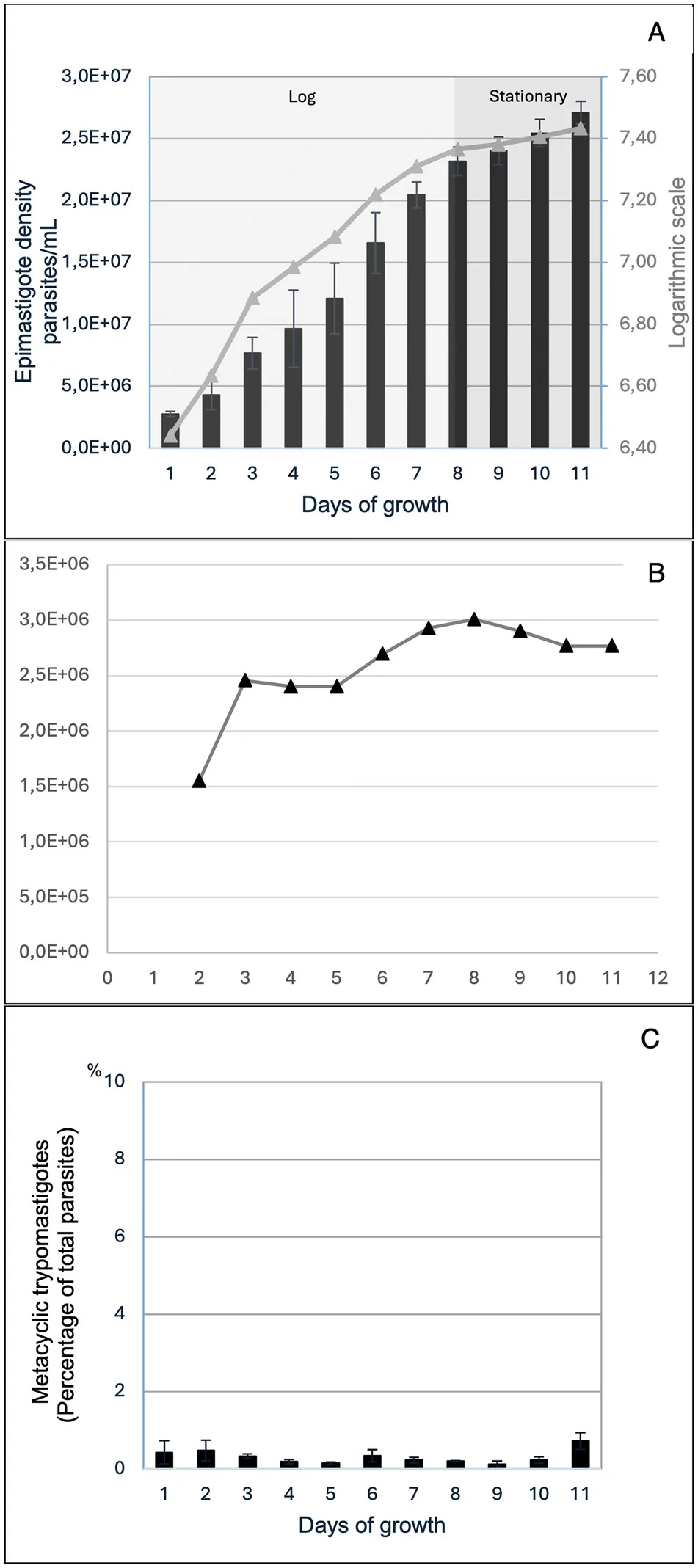
Analysis of axenic *T. cruzi* (Y strain) epimastigote growth over an 11-day period. (A) Parasite growth phases displayed on dual *y*-axes (left: linear scale, right: logarithmic scale). (B) Slope of correlation generated. (C) Metacyclic trypomastigote differentiation percentage during epimastigote culture. Parasite growth was measured daily throughout the experimental period.

The growth rate peaked on day 8 (Fig. 1B), then declined from days 9 to 11. Metacyclic trypomastigotes remained below 0.47% for most of the observation period, reaching 0.72 ± 0.21% on day 11 (Fig. 1C).

### Impact of initial parasite density on growth and measurement accuracy

A factorial analysis of variance ANOVA revealed that all tested variables significantly influenced parasite growth (*P* < .05).

Comparison of initial parasite densities (1 × 10^5^, 1 × 10^6^, and 5 × 10^6^ parasites/ml) showed that the lowest concentration (1 × 10^5^) exhibited reduced growth and greater variability relative to higher densities (Fig. 2A). The highest concentration (5 × 10^6^ parasites/ml) showed greater parasite growth; however, aggregate formation was observed at this density, hindering accurate parasite enumeration.

**Figure 2:**
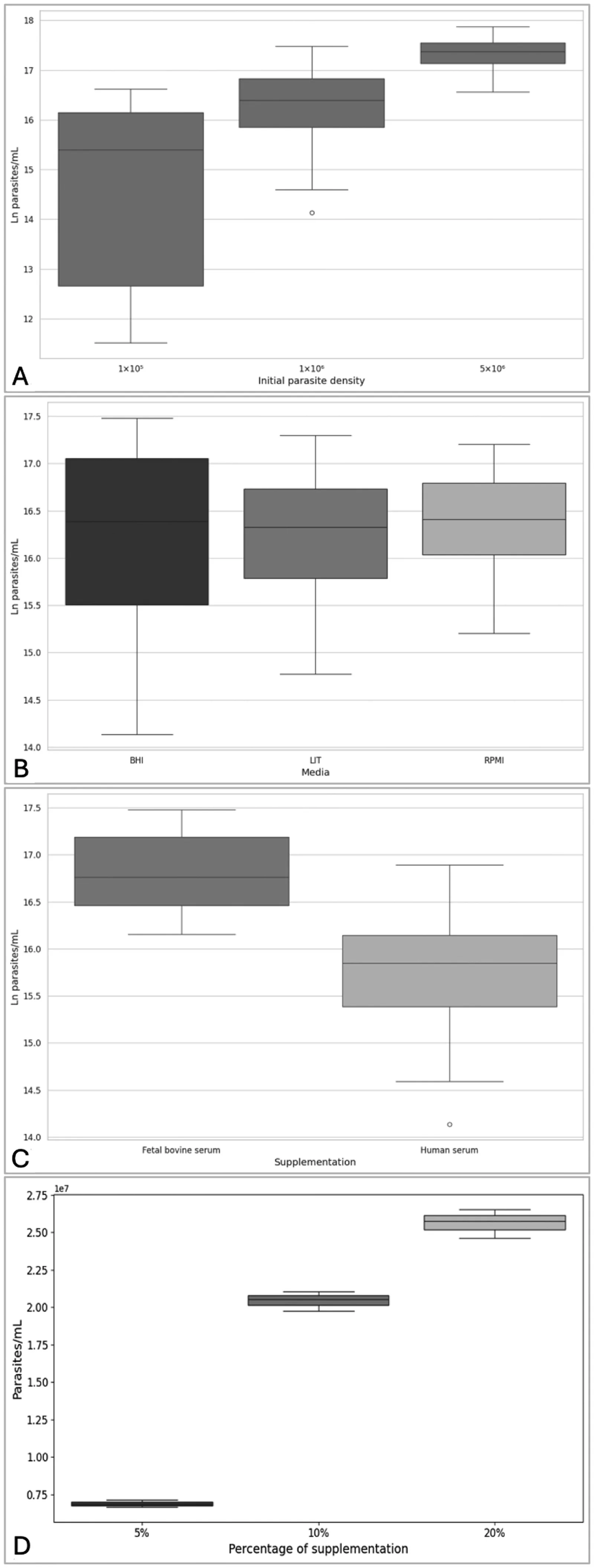
Boxplot showing the distribution of *T. cruzi* growth after 8 days of culture. (A) Grouped by initial parasite concentration (parasites/ml). (B) Grouped by culture medium type. (C) Grouped by serum supplementation type. (D) Grouped by FBS concentration (5, 10, and 20%)

### Culture medium and supplementation

After establishing the optimal starting inoculum, ANOVA showed no statistically significant differences in the *in vitro* growth of epimastigotes among the three media tested (*P* = .21; Fig. 2B).

Under optimized inoculum conditions, ANOVA revealed that only serum type significantly affected parasite growth (*P* = 1.53 × 10^−9^). The two serum types tested were fetal bovine serum (FBS) and human serum (HS). FBS supported higher parasite density than HS; post hoc Tukey analysis confirmed this difference, with FBS yielding 1.41-fold greater mean parasite density (*P* < .05; Fig. 2C).

To optimize FBS percentage, concentrations of 5%, 10%, and 20% were evaluated. Statistically significant differences in parasite density were observed among the tested percentages (*P* = 1.31 × 10^−7^; Fig. 2D). Tukey’s HSD test revealed a clear density hierarchy: 20% > 10% > 5%, with corresponding values of 2.56 × 10^7^, 2.05 × 10^7^, and 6.88 × 10^6^ parasites/ml, respectively. These results indicate a positive relationship between FBS concentration and parasite proliferation.

### Culture media replacement frequency

A 3-day medium replacement frequency sustained higher parasite growth from day 12 onward compared to 7-, 10-, and 15-day frequencies (Fig. 3). This schedule also maintained epimastigote percentages above 98%, with trypomastigote transition remaining below 2% until day 9—a value 10-fold lower than other frequencies at that time point. This maintenance of a predominantly epimastigote culture ensures uniformity in the desired parasite life stage for pharmacological testing (Fig. 3).

**Figure 3:**
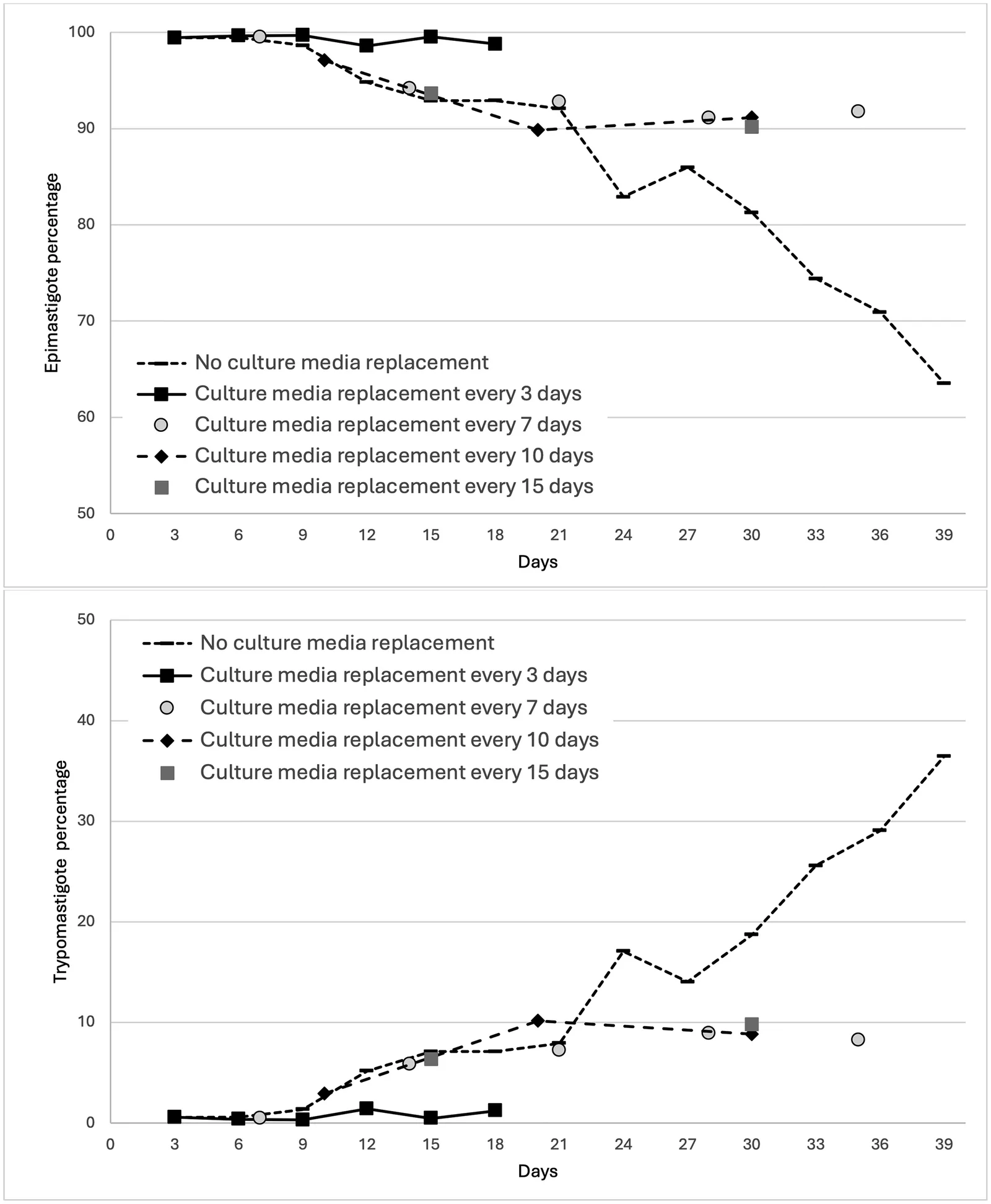
Time course showing the proportion of epimastigote and trypomastigote stages under different media replacement schedules

### Culture well plates

One-way ANOVA revealed no significant differences in parasite growth between plate types throughout the 11-day culture period (*P* > .05) (Fig. 4).

**Figure 4:**
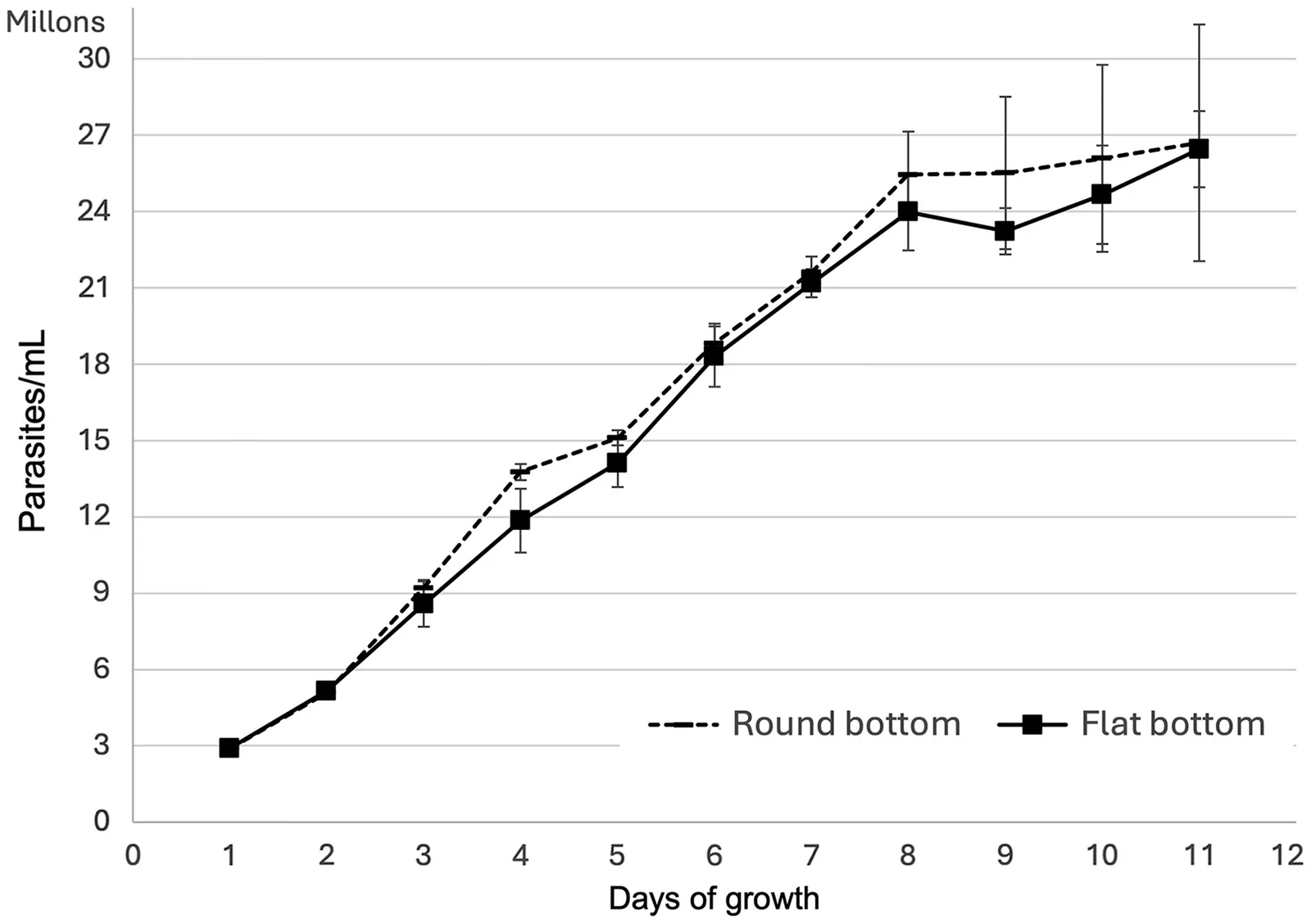
Parasite proliferation patterns in U-bottom and flat-bottom well plates. Values represent mean parasite concentration ± standard deviation

### Reference substances and controls

Both controls performed as expected: the vehicle control showed no growth inhibition, while the growth control (optimal conditions) maintained consistent proliferation, confirming assay reliability. The results demonstrated concentration-dependent inhibition of parasite growth, with Benznidazole yielding an IC_50_ of 16.61 ± 0.38 µM against *T. cruzi* under optimized conditions.

## Discussion

*Trypanosoma cruzi* epimastigotes exhibit stage-specific biological characteristics that may confer distinct druggable vulnerabilities. Although not the optimal primary model for mammalian infections, they serve as a valuable complementary screening tool, particularly given emerging evidence of their direct relevance to certain routes of human infection [10, 11]. Recent studies have demonstrated that newly differentiated epimastigotes (within 72 h post-differentiation) retain infectivity for vertebrate hosts [19], and the Brazilian consensus on CD recognizes their role in oral transmission and laboratory infections [10]—findings that collectively challenge the traditional view of epimastigotes as solely replicative forms.

The use of epimastigotes in the optimized assay should be interpreted within the biological complexity of the *T. cruzi* life cycle. Although this stage does not reproduce the intracellular environment of mammalian amastigotes, it retains core metabolic and redox systems that represent biologically relevant parasite vulnerabilities, including glycosomal pathways and the trypanothione-dependent antioxidant network [12]. Epimastigotes have therefore been widely used in phenotypic screening of anti-*T. cruzi* compounds. However, stage-specific differences in metabolism and physiology preclude interpreting activity against epimastigotes as a direct surrogate for activity against intracellular amastigotes. Consequently, activity identified in this model requires subsequent confirmation in intracellular amastigote assays, where host-parasite interactions and mammalian-stage biology are incorporated into the biological response.

Within this framework, the optimized epimastigote assay can be positioned as a scalable early-stage component of a *T. cruzi* drug discovery cascade rather than as a stand-alone surrogate for the intracellular stage. Its principal contribution is to provide a reproducible and operationally accessible first-pass filter for evaluating compound collections under harmonized conditions before advancing prioritized hits to more complex, resource-intensive models. This staged approach is particularly relevant for libraries preselected using physicochemical or computational criteria associated with drug-likeness, although such prefiltering does not establish intracellular exposure or antiparasitic selectivity. Compounds identified in the epimastigote assay should therefore undergo subsequent assessment of mammalian-cell cytotoxicity and activity against intracellular amastigotes, followed, where appropriate, by further optimization and *in vivo* evaluation. In this context, the optimized assay contributes to the early prioritization of active compounds while complementing, rather than replacing, higher-complexity models.

An additional advantage of the epimastigote model is its operational simplicity: parasites can be readily obtained, handled, and maintained *in vitro* without host cells or animal models, making the assay comparatively rapid and cost-effective for early-stage screening. A recent review of pharmacological sensitivity tests across *T. cruzi* life stages identified epimastigotes as the most frequently investigated form (47%), followed by amastigotes (29%) and trypomastigotes (24%) [20], underscoring their continued relevance as a complementary model in anti-*T. cruzi* pharmacological research [18].

*Trypanosoma cruzi* strain Y (*TcII*) is considered the strain of choice for drug efficacy evaluations, serving as a widely recognized experimental model for both *in vitro* and *in vivo* studies. Strain Y belongs to *TcII*, one of the four discrete typing units (DTUs: *TcI, TcII, TcV, TcVI*) associated with human infection [16], enhancing its clinical relevance for pharmacological research [21, 22].

### Parasite growth kinetics and treatment exposure time

The kinetics study revealed exponential growth until day 8, followed by a stabilization phase from day 9 onwards (Fig. 1A). This pattern is characteristic of epimastigote cultures, which exhibit an extended stationary phase during which cells exit the DNA synthesis cycle and either transform into metacyclic trypomastigotes or undergo cell death [23]. In this study, the proportion of metacyclic trypomastigotes remained low, increasing toward the end of the observation period (day 11), coinciding with nutrient depletion and suggesting a pre-adaptive stage for differentiation. Following metacyclogenesis, metacyclic trypomastigotes become capable of establishing infection in vertebrate hosts [24].

The growth kinetics observed in the present study are consistent with previously reported patterns. Ribeiro *et al*. [25] reported peak proliferation of *T. cruzi* strain Y epimastigotes between days 6 and 9, with stabilization beginning on day 7. Similarly, Santos *et al*. [24] observed the onset of the stationary phase between days 7 and 10 for *T. cruzi* strain Dm28c, peaking on day 8. In a multi-strain characterization including strain Y, Martinez-Diaz *et al*. [26] documented growth peaks exceeding 2.0 × 10^7^ parasites/ml between days 9 and 11, comparable to the present study (2.4–2.7 × 10^7^ parasites/ml). Furthermore, Querales *et al*. [27] described exponential growth of the *T. cruzi* EPm6 clone through day 8, coinciding with declining glucose levels and consistent with the predominantly glycolytic metabolism characteristic of epimastigote forms.

Treatment exposure time (TET)—the duration a test substance interacts with the parasite—directly influences the antimicrobial challenge presented to the parasite and can substantially affect experimental outcomes. TET-induced variability carries important implications, potentially increasing the risk of false positive or negative conclusions about a substance’s efficacy. The optimal TET must align with the parasite’s life cycle; *T. cruzi* Epimastigotes have a generation time of 24 h [28, 29]. However, pharmacological activity tests against *T. cruzi* epimastigotes often use incubation times ranging from 24 h to 11 days [18]. Prolonged exposure times may compromise optimal growth conditions, as extended cultures entering the stationary phase often exhibit nutrient depletion in media. A lack of nutrients can trigger transformation into metacyclic trypomastigotes [7, 30], resulting in a heterogeneous culture which may affect the accuracy of drug efficacy assessments.

In epimastigote-based pharmacological assays, the primary objective is to demonstrate inhibition of parasite proliferation. The TET should exceed 24 h to capture at least one full replication cycle. However, incubation periods longer than 8 days are methodologically unsuitable, as parasites enter the stationary phase (Fig. 1A), compromising the reliability of results. Accordingly, an optimal incubation time of 72 h is proposed, as this duration falls within the exponential growth phase, encompasses multiple replication cycles, and is consistent with protocols reported in the literature.

### Optimal infecting inoculum

The optimal initial inoculum density for pharmacological testing was determined by evaluating three parameters: parasite growth rate, density variability, and quantification reliability by microscopy. Accordingly, an inoculum of 1 × 10^6^ parasites/ml is recommended, as this concentration prevents parasite aggregate formation, minimizes data dispersion, and enables reliable quantification.

### Culture medium type and supplementation level

Comparison of culture media revealed no significant differences in *T. cruzi* epimastigote (Y strain) growth among RPMI, LIT, and BHI (*P* = .21; Fig. 2B). This finding aligns with Özbilgin *et al*. [31] who reported no significant differences in epimastigote growth across five media (RPMI 1640, Medium 199, SIM, Nutrient Broth, and BHI), over a 10-day period (*P* > .05).

A principal component analysis was conducted to assess culture medium performance, mapping parasite density (parasites/ml) across factor-level combinations in a factorial plane (Supplementary Fig. S1). RPMI was associated with trypomastigote-like differentiation, indicative of nutrient depletion and culture stress [27]. Accordingly, LIT and BHI were identified as optimal media for epimastigote growth, as both supported adequate parasite proliferation while maintaining homogeneous epimastigote populations, thereby limiting unwanted differentiation into trypomastigote-like forms.

Fetal bovine serum is widely used as a culture medium supplement in *in vitro* parasite culture for its ability to support cell proliferation and metabolic activity. FBS supplies essential components, including proteins, lipids, hormones, vitamins, minerals, and various growth and binding factors, while also buffering pH, stabilizing osmolarity, and inhibiting proteolytic activity. Despite these advantages, FBS presents notable limitations. Batch-to-Batch compositional variation, arising from inter-animal variability, can introduce experimental inconsistencies. Ethical concerns regarding animal welfare have been increasingly recognized, and FBS is costly, often requiring supplementation with additional acids, vitamins, and hormones to achieve optimal culture conditions [31, 32].

Figure 2D shows that 5% FBS concentration results in reduced parasite growth compared to higher concentrations. Villasuso *et al*. [33] examined delta-9-desaturase activity in *T. cruzi* epimastigote (Tulahuen strain) cultures grown in media containing 5% and 10% FBS and found that at 5% FBS, the activity of this enzyme—crucial for maintaining cell membrane integrity—decreased significantly, correlating with reduced parasite growth. Similarly, Jimenez *et al*. [34] demonstrated that FBS deprivation or reduction induced apoptosis in epimastigotes during exponential growth. Although FBS concentration positively correlates with parasite proliferation, the relationship is not linear. Economic analysis reveals that 20% FBS costs twice as much as 10% FBS but yields only 25% additional growth, illustrating diminishing returns at higher concentrations. Since the marginal growth benefit does not justify the increased expense, 10% FBS represents the optimal concentration for *T. cruzi* epimastigote cultures, balancing robust proliferation with cost–effectiveness.

### Culture media replacement frequency

Frequent medium replacement is essential for maintaining homogeneous axenic cultures of *Trypanosoma cruzi* and the target parasite form, in this case, epimastigotes. During prolonged incubation, epimastigotes undergo metacyclogenesis into metacyclic trypomastigotes: a process triggered by glucose depletion, typically occurring by day 11 in the Epm6 strain [27]. Consistent with this observation, Santos *et al*. [24] reported a progressive increase in the proportion of metacyclic trypomastigotes, with culture duration reaching 10.66% by day 10. Such shifts in parasite composition can significantly affect experimental outcomes and data interpretation. Frequent medium refreshing maintains a predominantly epimastigote population, ensuring experimental consistency and enabling accurate assessment of epimastigote-specific susceptibility. Under conditions of this study, the optimal medium replacement frequency is every 3 days. This higher replacement frequency likely contributes to a more stable growth environment for epimastigotes and prevents the accumulation of metabolic waste products that could otherwise alter parasite morphology or behavior.

### Culture well plates

A previous review examining the types of culture vessels used in *T. cruzi* epimastigote studies revealed substantial variability in methodology and reporting practices [18]. Analysis showed that 96-well plates were the most frequent format, followed by 24-well plates, with over 25% of studies omitting the plate type used altogether. A smaller proportion utilized alternative formats, such as tubes or culture boxes. The high proportion of studies lacking detailed information on culture vessel type is of particular concern, as insufficient methodological reporting undermines reproducibility—a cornerstone of scientific research.

Although well shape influences cell growth in culture, systematic comparison of biological model growth across culture plate geometries remains limited, resulting in suboptimal growth conditions and less reliable *in vitro* analyses [35]. Our study is the first to evaluate well geometry in *T. cruzi* epimastigote growth. Geometry is particularly important when alternative parasite quantification methods (such as colorimetric, turbidimetric, and fluorescence-based techniques) are used. This is driven by the technical requirements of plate readers, which perform more accurately with flat-bottomed wells [35].

Manual hemocytometer counting remains the most frequently reported approach for quantifying *T. cruzi* epimastigotes, though its limited scalability has prompted the adoption of alternative methods, including colorimetric, turbidimetric, and fluorimetric techniques, as well as automated cell-counting systems [18]. Although signal-based methods may enable higher-throughput analyses, they provide indirect or instrument-dependent readouts that require assay-specific optimization and validation. Turbidimetric measurements, for example, require calibration against known parasite densities and are susceptible to interference from culture conditions or cellular debris. Consequently, while automated or signal-based methods may improve throughput, direct manual counting remains an indispensable reference method for assessing parasite multiplication.

Both flat- and round-bottom well plates yielded comparable parasite growth and viability, demonstrating that the pharmacological model is independent of well geometry and allowing flexibility in experimental design, as either plate type produces equivalent results (Fig. 1).

### Reference substances and controls

Under optimized conditions, negative and growth controls established specifications for subsequent experiments. These growth levels were consistent with those observed at 72 h in the kinetics study. Additional controls confirmed that 0.04% DMSO did not affect parasite growth.

Vela *et al*. [20] conducted a meta-analysis assessing the *in vitro* susceptibility of different *T. cruzi* DTUs to benznidazole, reporting epimastigote IC_50_ values ranging from 2.79 µM to 208.18 µM. The authors attributed this broad variability to strain heterogeneity and methodological inconsistencies, even under the same parasite form, DTU, and incubation time [20]. A recent analysis of seven studies using 72-h treatment protocols reported a mean IC_50_ of 21.8 ± 10.8 µM [18]. Our result (16.61 ± 0.38 µM) falls within this range and shows improved precision, supporting the robustness of our experimental approach.

Optimization of *in vitro* pharmacological tests is essential for evaluating compounds with potential activity against *T. cruzi*. This process enhances screening efficacy and generates reliable, reproducible, and cost-effective data [7]. Optimized assays enable rigorous screening to identify genuinely promising candidates. These straightforward, rapid *in vitro* assays effectively filter candidates, reducing reliance on animal testing by reserving advanced *in vitro* assays and *in vivo* studies for compounds that demonstrate specific activity. This tiered approach improves drug discovery efficiency while aligning with ethical considerations in pharmacological research [7, 35].

## Conclusions

*Trypanosoma cruzi* epimastigotes (strain Y) exhibit continuous growth until day 8 of incubation. From day 9 onward, proliferation plateaued with the emergence of metacyclic trypomastigotes. To maintain cultures with >95% epimastigotes, the culture medium must be frequently replaced.

Optimized culture conditions for *T. cruzi* epimastigotes include LIT or BHI medium supplemented with 10% FBS, incubation at 28°C, and medium replacement every three days.

The optimal conditions for the *in vitro* drug susceptibility assay against epimastigotes of *T. cruzi* include: an inoculum of 1 × 10^6^ parasites/ml, 96-well plates, 72-h treatment exposure time, and benznidazole as the positive (active) control. These parameters ensure reproducibility across laboratories.

Under optimized conditions, growth (untreated parasites) and vehicle controls exhibit robust parasite proliferation, confirming assay reliability. The IC_50_ of benznidazole (16.61 ± 0.38 µM) aligns with previously reported values under similar conditions. These controls provide essential quality benchmarks for *in vitro* anti-trypanosomal drug screening.

## Supplementary Material

bpag051_Supplementary_Data

## Data Availability

Research data are available upon request.
